# Presence, Quantification, and Health Risk Assessment of Mycotoxins in Polyfloral Bee-Collected Pollen from Serbia

**DOI:** 10.3390/toxins18070303

**Published:** 2026-07-14

**Authors:** Slobodan Dolašević, Nikola Rokvić, Marko Jauković, Maja Petričević, Tanja Keškić, Marija Gogić, Aleksandar Ž. Kostić

**Affiliations:** 1 Institute for Animal Husbandry Belgrade–Zemun, Autoput Beograd-Zagreb 16, 11000 Belgrade, Serbia; mpetricevic@istocar.bg.ac.rs (M.P.); tkeskic@istocar.bg.ac.rs (T.K.); mgogic@istocar.bg.ac.rs (M.G.); 2 Scientific Institute of Veterinary Medicine of Serbia, Smolućska 11, 11000 Belgrade, Serbia; nikola.rokvic@nivs.rs; 3 Academy of Applied Studies Polytechnic, Katarine Ambrozić 3, 11050 Belgrade, Serbia; mjaukovic@politehnika.edu.rs; 4 Faculty of Agriculture, University of Belgrade, Nemanjina 6, 11080 Belgrade, Serbia; akostic@agrif.bg.ac.rs

**Keywords:** pollen, mycotoxins, ochratoxin A, aflatoxin B1, zearalenone, T-2/HT-2 toxin, deoxynivalenol, fumonisin

## Abstract

Bee-collected pollen (BCP), due to its valuable nutrient content, is considered a natural food suitable for human consumption. On the other hand, it should be noted that the rich nutritional value of BCP is also relevant in the context of potential contamination with fungal secondary metabolites, such as mycotoxins. In this study, a total of 26 pollen samples were collected from different locations in order to investigate the potential presence of mycotoxins. All BCP samples were obtained from beekeepers who produce pollen for further commercialization. The analyzed mycotoxins included ochratoxin A (OTA), aflatoxin B1 (AFB1), zearalenone (ZEN), T-2 toxin, HT-2 toxin, deoxynivalenol (DON), and fumonisins (FUMs), which were measured by direct competitive enzyme-linked immunosorbent assays. The obtained results indicated the presence of at least three toxins in all samples. AFB1, OTA, and ZEN were detected above the limit of quantification (LOQ) in all samples, whereas T-2 and HT-2 were below the LOQ in five samples and DON in four samples. FUMs were below LOQ in all samples. Due to the significant content of AFB1 determined in all samples, there is a potential risk for future consumers, demanding constant monitoring as well as improvement of BCP production.

## 1. Introduction

Mycotoxins are secondary metabolites produced by molds, which can be present in food through direct or indirect contamination [1]. Their presence in food causes public health concerns because they affect stability and quality of food [1,2,3]. Their occurrence is difficult to predict, posing significant challenges to food safety and public health [4,5,6]. Mycotoxigenic fungi, through their low molecular weight toxic metabolites, exhibit a wide range of specific disorders such as nephrotoxicity, hepatotoxicity, neurotoxicity, immunotoxicity, carcinogenicity, and teratogenicity [7]. Besides their harmful effects, an additional problem is that they are moderately stable at high temperatures and during other types of food-processing systems [8,9].

From the perspective of food safety, mycotoxin contamination of food is undesirable, and control is necessary to monitor their levels and keep them at the minimum possible level [10]. This issue is particularly relevant for vulnerable population groups, such as infants, where strict regulatory limits for contaminants are particularly important. Certain studies indicate the presence of mycotoxins in 98.3% of baby food samples, where 80% of the samples showed multiple mycotoxin contamination [11]. These studies specify that mycotoxin contamination is prevalent in a wide range of food matrices. The most commonly analyzed foods are cereals and cereal-based products, oilseeds, spices, and animal feeds [12,13,14]. On the other hand, studies on mycotoxin contamination in bee products remain limited [15,16,17].

Pollen, mostly referring to BCP, is widely known and recognized around the world for its nutritional and bioactive properties [18,19,20,21,22]. Pollen, as a nutritional resource, can be characterized by its diverse nutritional composition, containing a wide range of macro- and micronutrients [23,24]. The chemical composition and nutritional values of pollen grains make them a valuable functional food component [25,26]. The increasing consumption of bee products as nutritional supplements and functional foods highlights the importance of assessing potential contamination by mycotoxins [27]. Considering the above, it should be noted that before use, it is important to determine the presence of mycotoxins and the potential dietary exposure to them due to the potential hazard for humans.

Bearing in mind that honey bees store a large amount of food, this creates conditions that support the development of a diverse microbiota of bacteria and fungi [28]. Previous studies have suggested mutualistic coevolution between the honey bee and certain molds, which may explain the tolerance of honey bees to mycotoxins, especially aflatoxins, suggesting a long-standing evolutionary association with *Aspergillus* species [29,30]. In some studies, the presence of fungal spores may contribute to the nutritional value of pollen, compensate for nutritional imbalances of poor-quality pollen diets, and increase the longevity of honey bee workers [31]. Pollen is an important component of bee nutrition, serving primarily as a protein source. Its nutritional significance is particularly evident during specific periods of the honey bee life cycle, when it serves as the primary source of proteins, lipids, vitamins, and minerals essential for larval development [32].

It is well documented that some beehive-derived products, such as honey and propolis, exhibit antibacterial and antifungal properties, acting as strong antimicrobial agents with a wide range of effects [33,34]. In contrast, BCP, due to its nutrient richness, provides a favorable medium for the presence and activity of bacteria, molds, and yeasts, which is why bees biotransform pollen into bee bread, leading to changes in its microbial ecosystem and enhancing its overall stability against microbial spoilage [35]. In particular, BCP is suitable for the growth of molds due to its nutritional values, favorable pH, and a_w_, as well as multiple routes of contamination with mycotoxins [36]. The natural mycobiota occurring in BCP can influence its safety and quality [37]. The level of contamination differs with geographic location and the production of certain mycotoxins is influenced by environmental conditions or by agricultural processing methods of production and storage [38]. BCP may become contaminated from the environment by pesticides, toxic elements, metalloids, and mycotoxins. And because there is currently no European standard regulation, as well as a lack of specific regulations for pollen, its use could pose food safety risks [39,40,41].

To the best of our knowledge, this is the first comprehensive study to investigate the occurrence of the selected multiple mycotoxins in BCP in Serbia, aiming to fill existing data gaps, as current data on the quantity and types of mycotoxins remain underexplored. While previous studies conducted in Serbia have focused exclusively on the occurrence of aflatoxin B1 [42,43], the present study is the first to simultaneously assess seven mycotoxins in BCP and to evaluate their potential impact from a human health risk assessment perspective. In addition, this study provides a broad screening of BCP contaminants which is important due to its application as a functional food, providing new data on the mycological quality of BCP collected from different localities in Serbia, as well as the potential health risks associated with its consumption. In this way, the current study will provide baseline data for the region and fulfill the existing scientific gap for the Balkans region.

## 2. Results

This study focused on analyzing the mycotoxins present in pollen collected from 26 samples originating from different geographical locations. In Table 1, the occurrence and concentrations of the analyzed mycotoxins in all polyfloral pollen samples are presented. The concentrations are expressed in µg/kg (ppb). All fields marked in red represent samples in which mycotoxins were detected. All fields marked in green represent samples in which mycotoxins were below the detection limit. In all pollen samples, OTA and AFB1 as well as ZEN were found, whereas T-2/HT-2 and DON were detected in most samples. Of all the analyzed mycotoxins, only FUM was below the limit of detection in all samples (Table 1).

Complete absence of mycotoxins was not recorded in any of the tested samples. In the analyzed pollen, all samples had at least three different mycotoxins. As many as 65% of all samples were positive for five different mycotoxins (Table 2).

**Figure 1:**
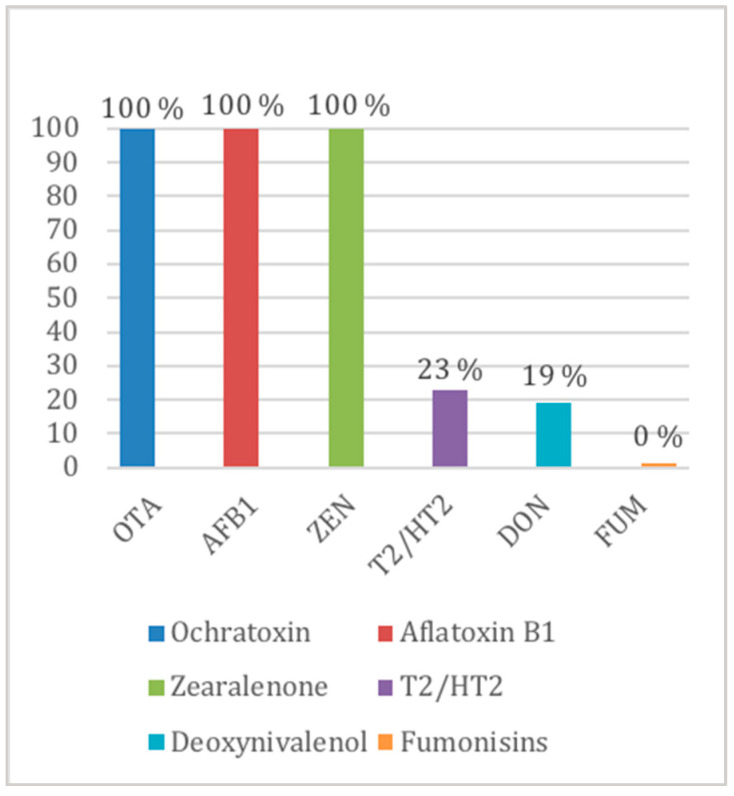
The proportion (share) of individual mycotoxins in the tested pollen.

**Figure 2:**
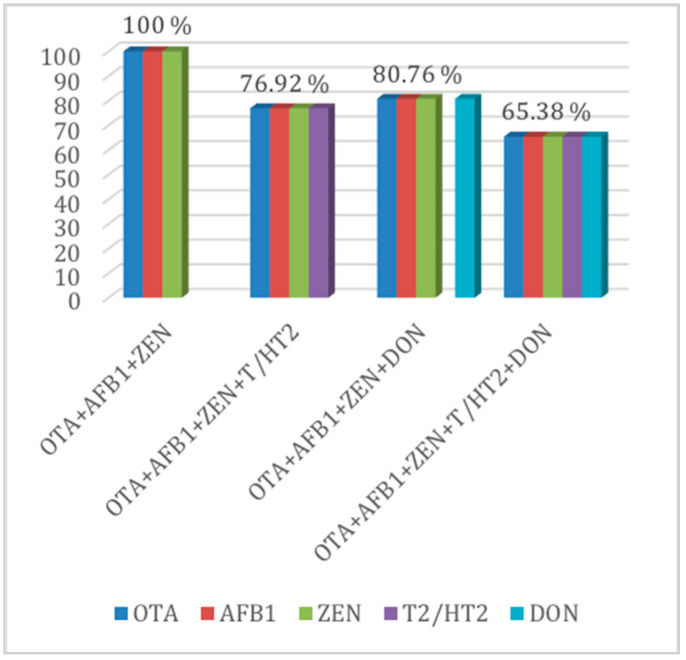
The proportion of combined mycotoxins in the tested pollen.

Figure 1 shows the percentage share of individual mycotoxins and their total share in all analyzed samples. Co-occurrence of mixed mycotoxins is shown in Figure 2, which confirms the multiple presence of different mycotoxins in individual pollen samples. Different combinations of two or more mycotoxins are reported in previous studies, where some findings identify even more than seven different mycotoxins [44].

### Mycotoxins Exposure and Health Risk Assessment

Results for mycotoxin exposure are presented in Table 3.

Based on the obtained results, there are significant differences among both the examined samples and the monitored mycotoxins. A higher exposure pattern was observed for ZEN (13.7–115.4 ng/kg b.w. per day) and DON (0–92.3 ng/kg b.w. per day) compared to OTA, AFB1 as well as T2/HT2 while samples 15, 24, and 25 stood out with the highest total mycotoxin exposure. Among samples the lowest exposure was observed for samples 17 and 26 due to absence of T2/HT2 and DON.

Based on daily exposure calculation, further assessment of health risk for humans has been performed by calculation of the hazard quotient (HQ) / hazard index (HI) values for all examined mycotoxins as well as margin of exposure (MOE) value for AFB1 due to its high toxicity [45]. The obtained results are presented in Table 4.

All HQ values were below 1, while MOE values for AFB1 were in the range between 120.6 (sample 22) and 703.5 (sample 6). Based on the total sum HI values obtained were in the range between 7.22 (sample 17) and 43.1 (sample 24).

## 3. Discussion

Mycotoxicological analysis revealed that out of 26 BCP samples examined in this study, OTA, AFB1, and ZEN had the highest occurrence rate (100%), followed by DON (81%), and T2/HT2 (77%), while FUM was below the limit of detection in all analyzed samples (Table 1). The obtained results are in accordance with the results of Kačaniová et al. [46], where they analyzed the concentration of mycotoxins in Slovak samples of rape and dried poppy pollen. According to these authors, most parameters showed similar values compared to the obtained one, where all tested mycotoxins in poppy pollen were detected, except FUM. While the values of most measured mycotoxins are comparable, a significant difference appears in the level of T-2 toxin, which in their study [46] was considerably higher compared to results from the current study. Absolute occurrence rate for AFB1 in samples collected in Serbia was also observed in previous studies by Kostić et al. [42] and Petrović et al. [43], with slightly higher average values. Extremely high occurrence rate of AFB1 (98.75%) was also reported by Carrera et al. [47] in their study but with a lower maximum concentration (5.3 µg/kg). On the other hand, the occurrence rate of OTA, ZEN, and DON were significantly lower, and their presence was quantified in 28.75%, 55%, and 66.25% of the analyzed samples, respectively. Maximum concentrations of DON, ZEN, and OTA were 135 µg/kg, 622 µg/kg and 7.8 µg/kg, respectively. Nuvoloni et al. [48], also reported a 100% occurrence rate of AFB1 with significantly wider range of concentrations (5.2–34.4 µg/kg), while the occurrence rate of DON (86.2%) in their study was slightly higher than in current study, with lower maximum concentration reported (179.7 μg/kg). AFB1 was reported to be the most frequent by Keskin et al. [27] as well, however with a significantly lower occurrence rate (25–30%). A low to moderate occurrence rate of OTA (23.5%) was also reported by Vegh et al. [41]. The frequency rate of DON (85–90%), reported by Sinkevičiene et al. [49], was slightly higher; however, the concentration range (47–120 µg/kg) was significantly lower.

On the other hand, FUM mycotoxins are predominantly found in cereals, primarily in maize and maize-based products [50], and their presence was not detected in the examined pollen samples, while certain studies have reported the detection of some amounts of FUM [46]. Other studies have also shown that all analyzed pollen samples had quantifiable levels of mycotoxins [47]. By comparison with other bee products, mycotoxins such as DON, T-2 toxin, HT-2 toxin, and OTA were also detected in other bee products [27].

A very high rate of mycotoxin co-contamination was observed (Table 2 and Figure 2), with co-occurrence of the three major mycotoxins (AFB1, DON, and ZEN) detected in all analyzed samples. Carrera et al. [47] in their study reported the co-occurrence of same mycotoxins, however in a significantly lower number of analyzed samples (32.5%), which can be caused by higher number of the analyzed samples.

Co-occurrence of all five detected mycotoxins in all analyzed samples was also very high (65.4%). Kačaniová et al. [46] also reported a very high level of co-occurrence of 5 different mycotoxins in rape bee pollen and poppy bee pollen samples and 6 different mycotoxins in sunflower bee pollen samples.

When it comes to pollen, it is difficult to obtain comprehensive information on regulatory limits and permitted maximum levels at the global level, as multiple possible exposure scenarios and combinations exist with respect to the type of food products, population groups, and consumption patterns [51]. The amount of toxins in food is also directly related to the amount of consumption of contaminated food.

Maximum allowed concentrations for mycotoxins in pollen are not set. Some authors recommend that maximum allowed concentration for AFB1 should be set at 2 µg/kg [52].

It is difficult to define a safe intake level for mycotoxins, while the mean lower bound (LB) can be used, as it represents the lowest possible exposure based on measured concentrations. According to Schrenk et al. [53], the values of AFB1 for younger age groups showed that the mean LB exposure ranged from 0.08 to 1.78 ng/kg body weight per day, and the mean upper bound (UB) exposure ranged from 0.58 to 6.95 ng/kg body weight per day, for adults, the mean LB exposure ranged from 0.22 to 0.49 ng/kg body weight per day, and the mean UB exposure ranged from 1.35 to 3.25 ng/kg body weight per day. Certain reports indicate that the average daily intake of AFB1 ranges from 2 ng to 77 ng in humans [54].

By analyzing our obtained values, we estimated the intake of mycotoxins potentially through the consumption of the tested pollen samples. For example, the average value of AFB1 in pollen is 5.58 μg/kg, which means that one gram of pollen contains 5.58 ng. This amount, for a person weighing 70 kg, who would consume only one gram of pollen daily, represents around 0.08 ng/kg body weight per day. According to the European Food Safety Authority, if a person consumed a larger amount of food with this concentration of mycotoxin, there would be a risk to the safety of such food [53]. Additionally, according to this assessment, EFSA relies more on a MOE approach rather than defining a fixed tolerable intake value.

By analyzing the results for mycotoxin exposure (except for AFB1) due to their presence in bee-collected pollen samples based on the obtained HQ/HI values (Table 4), it can be observed that all obtained HQ values were below 1 (i.e., 100% of HQ values were below 1). This was consistent across all samples. This suggests that daily consumption of BCP as a food supplement will not cause an elevated risk. However, it should be pointed out that exposure is a cumulative factor that can be additionally increased by the consumption of other foodstuffs that may also be contaminated with the same toxins as well as by simultaneous presence of different mycotoxins in different foods [55]. In contrast, this was different in the case of AFB1 exposure assessment.

To assess health risk for consumers triggered by AFB1 presence in BCP samples, MOE values have been calculated. According to the recommendation, MOE values lower than 10,000 are considered as potentially dangerous [45]. Since all obtained MOE values were below the suggested threshold value, it can be speculated that AFB1 presence in BCP samples can cause risk for potential consumers. This is a worrying result due to high toxicity and carcinogenicity of AFB1 for humans [56]. However, the given observation, once again, confirms the importance of simultaneously conducting both microbiological and mycotoxicological analysis of BCP, as is already suggested in the literature [36].

In a similar way, HI calculation for other mycotoxins allows us to assess potential risk for consumers caused by multitoxins presence in BCP. With HI values significantly lower than 100%, it can be assumed that there is no substantial risk for potential consumers [45]. However, in some cases and for some samples, consumption of contaminated pollen, as a food supplement, can significantly increase risk with parallel consumption of some regular foods contaminated with mycotoxins [57]. Recently Carrera et al. [47] have conducted a detailed survey on presence of mycotoxins in commercial BCP samples obtained from 28 European countries with results also pointing out some potential risk for consumers due to multiple presence of these hazardous substances, in particular AFB1 as carcinogenic. In this sense, the present study provides data for Serbia, which was not included in the aforementioned study, thereby contributing to the existing literature.

Overall, the risk assessment indicates that BCP, based on the obtained HI values below the acceptable threshold, has a low probability of causing adverse health effects related to non-carcinogenic mycotoxins. However, the presence of AFB1 requires particular attention due to its carcinogenic potential and the need for continuous monitoring of BCP intended for human consumption. Cumulative exposure from other contaminated food sources should also be considered when evaluating the overall consumer risk. It should be noted that this risk assessment represents an estimation based on average consumption values and detected mycotoxin concentrations, while individual exposure may vary depending on consumption habits. Therefore, although BCP consumption under the assumed conditions does not represent a significant risk for most analyzed mycotoxins, continuous monitoring of mycotoxin contamination should be considered due to the susceptibility of pollen to contamination. Further investigations are also needed to contribute to a better understanding of this issue and to ensure consumer safety.

## 4. Conclusions

In the current study, the occurrence of multiple mycotoxins in the analyzed pollen from various locations was presented. In all collected pollen samples, at least three mycotoxins were present, while in 65% of the pollen samples, five or more mycotoxins were detected. These results suggest previous contamination of BCP with mycotoxins, indicating prior fungal activity. However, the current study did not make conclusions for how to ensure that pollen is safe for consumption or what the permitted limits are for the amounts of mycotoxins in BCP, as they are not regulated. Also, it cannot be specified at which stage of BCP production mycotoxin formation occurred. There is a possibility of subsequent contamination through contact with the bees or during storage in the pollen trap at the time of collection. A third option is that fungal activity occurred during its processing in the production facility up to the moment of analysis (even though it was stored in a freezer). Accordingly, in the future, further research is planned in order to resolve the aforementioned uncertainties. The levels of mycotoxins in pollen need to be more clearly defined in legal and regulatory terms in order to enable the establishment of clear recommendations for its safe use and consumption. Although the present study provides important baseline data on the occurrence of mycotoxins in BCP from Serbia, it is limited by the study sample size. Future studies should include a larger number of samples collected over multiple seasons and regions, together with more comprehensive statistical analyses (such as multivariate analyses) to better evaluate the factors influencing mycotoxin occurrence. A limitation of the present study is that the obtained ELISA results should be considered preliminary, as they were not further confirmed by chromatographic methods. Future studies should include confirmatory analysis using LC-MS/MS to further validate aflatoxin occurrence in BCP samples and provide a more comprehensive assessment of their occurrence. Continuous monitoring and the implementation of appropriate quality control measures are recommended to ensure the safety of BCP intended for human consumption.

## 5. Materials and Methods

### 5.1. Sampling

**Figure 3:**
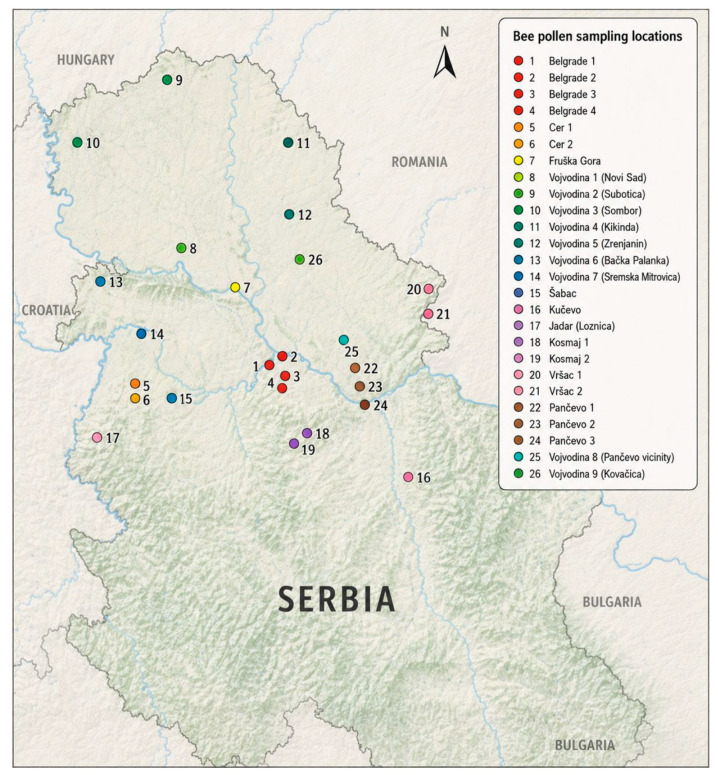
Bee pollen sampling locations.

BCP was obtained by using pollen traps placed at hive entrances and dried by beekeepers engaged in commercial pollen production. All 26 pollen samples were collected from different locations across Serbia (Figure 3) by local beekeepers.

Each sample contained 30–50 g of pollen, packaged in small labeled bags. Pollen samples were collected during the spring and summer seasons of 2025. In order to prevent additional contamination, all samples were stored and maintained under controlled freezing conditions at the Institute for Animal Husbandry (Belgrade, Serbia) until further analysis. Although palynological analysis was not performed, all BCP samples were characterized as polyfloral due to differences in colors of the pollen grains, indicating different botanical origins.

### 5.2. Mycotoxicological Analysis

Considering that all tested mycotoxins are of low molecular weight, their detection was performed using the immunochemical method, the competitive enzyme-linked immunosorbent assay (ELISA), which provides highly sensitive and reliable quantification and detection even at low toxin concentration. Each sample was analyzed once, and the reported results represent single measurements. The analysis of mycotoxins was conducted at the Polihem Laboratory, Academy of Applied Studies Polytechnic (Belgrade, Serbia). The procedure consisted of the initial homogenization of the sample (obtaining subsamples), followed by extraction with an appropriate solvent and their filtration according to the instructions of the respective kit manufacturers. ELISA commercial test kits were used to quantify the concentration of mycotoxins, and all tests were validated according to the Eurachem Guide [58], which was also used for the determination of the limit of detection (LOD) during method validation. For each mycotoxin analyzed, a different and specific ELISA kit was used. Sample preparation and validation parameters were as follows:

- For the analysis of OTA, commercial RIDASCREEN^®^ by R-Biopharm (Darmstadt, Germany) Ochratoxin A 30/15 kit–R1312 was used. Limit of detection was set at 0.3 ppb with measuring range from 0.3 to 30 ppb. Recovery ranged from 94 to 108%, and standard deviation was 12.34%. Samples were extracted using the RIDASCREEN ECO extractor.
- For the analysis of AFB1, commercial RIDASCREEN^®^ Aflatoxin B1 30/15 kit–R1211 was used. Limit of detection was set at 1 ppb with measuring range from 1 to 50 ppb. Recovery ranged from 79 to 108%, and standard deviation was 15.68%. Samples were extracted with 70% methanol prior to ELISA analysis.
- For the analysis of ZEN, commercial RIDASCREEN^®^ FAST Zearalenon SC kit–R21444 was used. Limit of detection was set at 5 ppb with measuring range from 5 to 1000 ppb. Recovery ranged from 88 to 106%, and standard deviation was 11.34%. Samples were extracted with 70% methanol prior to ELISA analysis.
- For the analysis of T-2/HT-2 toxin, commercial RIDASCREEN^®^ T2/HT-2 Toxin kit–R3805 was used. Limit of detection was set at 10 ppb with measuring range from 10 to 360 ppb. Recovery was 94%, and standard deviation was 12.84%. Samples were extracted with 70% methanol.
- For the analysis of DON, commercial RIDASCREEN^®^ FAST DON SC kit–R23414. Limit of detection was set at 75 ppb with measuring range from 75 to 6000 ppb. Recovery was from 93 to 101%, and standard deviation was 15.84%. Samples were extracted with distilled water.
- For the analysis of total FUM, commercial RIDASCREEN^®^ Fumonisin ECO kit–R3411. Limit of detection was set at 25 ppb with measuring range from 25 to 2000 ppb. Recovery was from 87 to 120%, and standard deviation was 10.84%. Samples were extracted using the RIDASCREEN ECO extractor.

The ELISA microplate readers used in the study were Biotek Agilent 800TS, with optical density measured at a wavelength of 450 nm. The concentration of mycotoxins was calculated using RIDASOFT Win/RIDA SOFT Win.net software version 1.9.1.—Art. Nr. R9996. All obtained results are expressed as µg/kg of dry weight of BCP samples.

### 5.3. Statistical Analysis

Descriptive statistics, including mean, minimum, maximum, variance, and standard deviation, were calculated using the open-source statistical software R (version 2.11.1). For each mycotoxin, SDs were calculated using the sample standard deviation formula with (*n* − 1) denominator based on the values obtained from all analyzed BCP samples. Each BCP sample was analyzed once. Measurements were not performed in duplicate or triplicate.

### 5.4. Health Risk Assessment

Exposure assessment was performed by combining the measured mycotoxin concentrations in BCP with estimated daily intake of pollen, taking into account average consumption rates for adult consumers. Namely, it was suggested that consumption of 20–40 g of BCP daily is fully safe for adults, as it was suggested in the literature [22]. The obtained exposure values were then compared with established toxicological reference values. In order to assess potential risk for consumer health, a risk assessment was performed with calculation and determination of the following parameters: exposure values (for each mycotoxin) and risk assessment through MOE calculation for AFB1 (as a carcinogen) as well as HI calculation for other non-carcinogenic mycotoxins, as it is described in detail in previously published research [45]. Based on this exposure assessment, risk characterization was performed using the MOE approach for aflatoxin B1 and the HI for non-carcinogenic mycotoxins. It should be noted that MOE values do not directly quantify risk but rather indicate the level of health concern, with lower MOE values corresponding to a higher level of concern. In numerical terms, MOE values above 10,000 are generally considered to represent a low risk to human health and a low public health concern. Conversely, MOE values below 10,000 suggest a higher level of public health concern. Unlike the MOE approach, an HI value exceeding 100% indicates that the cumulative exposure to multiple mycotoxins has surpassed the acceptable safety threshold [45].

## Figures and Tables

**Table 1:** Presence of individual mycotoxins (μg/kg dry weight) in all tested BCP samples (ochratoxin A–OTA, aflatoxin B1–AFB1, zearalenone–ZEN, T-2/HT-2 toxins–T-2/HT-2, deoxynivalenol–DON, fumonisins–FUMs); standard deviations (SD) were calculated using the sample standard deviation formula (divided by *n* − 1) from all analyzed BCP samples and represent the variability among samples.

Sample	OTA	AFB1	ZEN	T2/HT2	DON	FUM
1.	1.20	4.29	47.81	<10	157	<25
2.	1.25	3.88	50.20	<10	140	<25
3.	1.47	5.44	86.52	15.70	133	<25
4.	1.90	4.75	116.71	12.01	242	<25
5.	1.85	4.50	122.90	11.20	255	<25
6.	1.54	1.99	73.31	16.58	323	<25
7.	1.74	5.51	101.77	18.77	204	<25
8.	1.80	4.90	111.24	20.20	198	<25
9.	1.98	5.02	124.50	<10	202	<25
10.	1.64	4.25	101.20	<10	154	<25
11.	1.59	3.67	75.97	18.50	<75	<25
12.	1.74	3.76	126.16	10.84	<75	<25
13.	2.25	4.16	134.18	15.20	115	<25
14.	3.02	8.62	397.18	23.80	84	<25
15.	3.93	9.06	395.96	15.47	164	<25
16.	4.90	7.50	280.20	18.20	212	<25
17.	1.15	4.07	85.35	<10	<75	<25
18.	1.90	5.10	88.20	12.20	111	<25
19.	1.22	9.70	168.03	13.72	115	<25
20.	2.76	5.59	372.59	13.82	187	<25
21.	1.88	5.14	350.80	10.80	198	<25
22.	2.30	11.61	212.75	13.45	188	<25
23.	1.69	6.88	152.13	13.81	<75	<25
24.	3.80	6.07	403.90	16.70	151	<25
25.	3.45	5.02	390.20	15.20	165	<25
26.	2.98	4.66	280.34	<10	<75	<25
Mean	2.19	5.58	186.54	12.25	142.23	-
Lowest value	1.15	1.99	47.81	<10	<75	-
Highest value	4.9	11.61	403.9	23.8	323	-
Var	0.92	4.60	15,617.04	48.16	7457.47	-
SD	0.96	2.14	124.97	6.94	86.36	-

**Table 2:** The percentage of multiple mycotoxins in individual samples.

Category	Number of Samples	Percentage
≥3 mycotoxins	26	100%
≥4 mycotoxins	24	92.3%
≥5 mycotoxins	17	65.4%
≥6 mycotoxins	0	0%

**Table 3:** Mycotoxins exposure (ng/kg b.w. per day) for adults via daily consumption of BCP; N/A–not assessed because mycotoxin content was below LOQ/LOD; SD were calculated using the sample standard deviation formula (divided by *n* − 1) from all analyzed BCP samples and represent the variability among samples.

Sample	Exposure				
	OTA	AFB1	ZEN	T2/HT2	DON
1	0.343	1.226	13.66	N/A	44.857
2	0.357	1.109	14.343	N/A	40
3	0.420	1.554	24.72	4.486	38
4	0.543	1.357	33.346	3.431	69.143
5	0.529	1.286	35.114	3.2	72.857
6	0.440	0.569	20.946	4.737	92.286
7	0.497	1.574	29.077	5.363	58.286
8	0.514	1.400	31.783	5.771	56.571
9	0.566	1.434	35.571	N/A	57.714
10	0.469	1.214	28.914	N/A	44
11	0.454	1.049	21.706	5.286	N/A
12	0.497	1.074	36.046	3.097	N/A
13	0.643	1.189	38.337	4.343	32.857
14	0.863	2.463	113.48	6.8	24
15	1.123	2.589	113.131	4.42	46.857
16	1.400	2.143	80.057	5.2	60.571
17	0.329	1.163	24.386	N/A	N/A
18	0.543	1.457	25.2	3.486	31.714
19	0.349	2.771	48.009	3.920	32.857
20	0.789	1.597	106.454	3.949	53.429
21	0.537	1.469	100.229	3.086	56.571
22	0.657	3.317	60.786	3.843	53.714
23	0.483	1.966	43.466	3.946	N/A
24	1.086	1.734	115.4	4.771	43.143
25	0.986	1.434	111.486	4.343	47.143
26	0.851	1.331	80.097	N/A	N/A
Mean	0.626	1.595	53.297	4.374	50.313
Lowest value	0.329	0.569	13.66	3.086	24
Highest value	1.4	3.317	115.4	6.8	92.286
Var	0.071	0.361	1225.824	0.892	237.925
SD	0.268	0.601	35.012	0.944	15.425

**Table 4:** Health risk assessment for quantified mycotoxins for adults via consumption of BCP. * Provisional maximum tolerable daily intake (PMTDI): 14 ng/kg body weight (b.w.) per day (OTA); 500 ng/kg b.w. per day (ZEN); 60 ng/kg b.w. per day (T2/HT2) 1000 ng/kg b.w. per day (DON). ** BMDL 10 = 400 ng/kg (AFB1); N/A–not assessed because mycotoxin content was below LOQ/LOD. Mean, SD, and variance were calculated using only samples in which the mycotoxin was detected. N/A samples were excluded from these calculations.

Sample	OTA	AFB1	ZEN	T2/HT2	DON	
	HQ *	MOE **	HQ	HQ	HQ	HI (%)
1	0.024	326.340	0.027	N/A	0.0449	9.67
2	0.026	360.825	0.029	N/A	0.04	9.42
3	0.030	257.353	0.049	0.075	0.038	19.22
4	0.039	294.737	0.067	0.057	0.069	23.18
5	0.038	311.111	0.070	0.053	0.073	23.42
6	0.031	703.518	0.042	0.079	0.092	24.46
7	0.036	254.083	0.058	0.089	0.058	24.13
8	0.037	285.714	0.064	0.096	0.057	25.31
9	0.040	278.884	0.071	N/A	0.058	16.93
10	0.033	329.412	0.058	N/A	0.044	13.53
11	0.032	381.471	0.043	0.088	N/A	16.40
12	0.036	372.340	0.072	0.052	N/A	15.92
13	0.046	336.538	0.077	0.072	0.033	22.78
14	0.062	163.413	0.227	0.113	0.024	42.59
15	0.080	154.525	0.226	0.074	0.047	42.70
16	0.100	186.667	0.160	0.087	0.061	40.74
17	0.023	343.980	0.049	N/A	N/A	7.22
18	0.039	274.510	0.050	0.058	0.032	17.90
19	0.025	144.330	0.096	0.065	0.033	21.91
20	0.056	250.447	0.213	0.066	0.053	38.85
21	0.038	272.374	0.200	0.051	0.057	34.68
22	0.047	120.586	0.122	0.064	0.054	28.63
23	0.034	203.488	0.087	0.066	N/A	18.72
24	0.078	230.643	0.231	0.080	0.043	43.10
25	0.070	278.884	0.223	0.072	0.047	41.29
26	0.061	300.429	0.160	N/A	N/A	22.10
Mean	0.045	285.215	0.107	0.073	0.050	24.8
Lowest value	0.023	120.586	0.027	0.051	0.024	7.22
Highest value	0.1	703.518	0.231	0.113	0.092	43.1
Var	0.0004	11,925.763	0.005	0.0002	0.0002	117.74
SD	0.019	109.205	0.07	0.016	0.015	10.85

## Data Availability

The original contributions presented in this study are include in the article. Further inquiries can be directed to the corresponding author.

## Abbreviations

The following abbreviations are used in this manuscript:

BCP	Bee-collected pollen
OTA	Ochratoxin A
AFB1	Aflatoxin B1
ZEN	Zearalenone
DON	Deoxynivalenol
FUM	Fumonisins
LOQ	Limit of quantification
HQ	Hazard quotient
HI	Hazard index
MOE	Margin of exposure
LB	Lower bound
UB	Upper bound
b.w.	Body weight
